# Enhanced Vitellogenesis in a Whitefly via Feeding on a Begomovirus-Infected Plant

**DOI:** 10.1371/journal.pone.0043567

**Published:** 2012-08-24

**Authors:** Jian-Yang Guo, Sheng-Zhang Dong, Xiu-ling Yang, Lu Cheng, Fang-Hao Wan, Shu-Sheng Liu, Xue-ping Zhou, Gong-Yin Ye

**Affiliations:** 1 Ministry of Agriculture Key Laboratory of Agricultural Entomology, Institute of Insect Sciences, Zhejiang University, Hangzhou, China; 2 College of Life Sciences, China Jiliang University, Hangzhou, China; 3 State Key Laboratory of Rice Biology, Institute of Biotechnology, Zhejiang University, Hangzhou, China; 4 State Key Laboratory for Biology of Plant Diseases and Insect Pests, Institute of Plant Protection, Chinese Academy of Agricultural Sciences, Beijing, China; New Mexico State University, United States of America

## Abstract

**Background:**

The MEAM1 (B biotype) *Bemisia tabaci* (Gennadius) is one of the most widespread and damaging whitefly cryptic species. Our previous studies discovered that the MEAM1 whitefly indirectly benefits from interactions with the tomato yellow leaf curl China virus (TYLCCNV) via accelerated ovarian development and increased fecundity. However, the physiological mechanism of begomoviruse-infected plants acting on the reproduction of the insect vector was unknown.

**Methodology/Principal Findings:**

Biochemical and molecular properties of vitellogenin (Vg) and vitellin (Vt) were characterized in the MEAM1 whitefly. In addition, kinetics of Vt levels in ovary and Vg levels in hemolymph in different stages were detected using a sandwich ELISA. The level of hemolymph Vg increased rapidly after eclosion. A significantly higher level of hemolymph Vg and ovary Vt were observed in whiteflies feeding on virus-infected tobacco plants than those feeding on uninfected plants. In order to detect the levels of Vg mRNA transcription, complete vitellogenin (Vg) mRNA transcripts of 6474 bp were sequenced. Vg mRNA level in whiteflies feeding on virus-infected plants was higher than those feeding on uninfected plants. However, virus-infection of the whiteflies per se, as demonstrated using an artificial diet system, did not produce significant changes in Vg mRNA level.

**Conclusions/Significance:**

In MEAM1 whitefly, increased levels of both vitellin and vitellogenin as well as increased transcription of Vg mRNA are associated with feeding on begomovirus-infected plants, thus providing a mechanism for accelerated vitellogenesis. We conclude that MEAM1 whitefly profits from feeding on begomovirus-infected plants for yolk protein synthesis and uptake, and thereby increases its fecundity. These results not only provide insights into the molecular and physiological mechanisms underlying the elevated reproduction of a whitefly species through its association with a begomovirus-infected plant, but also provide a better understanding of the molecular mechanisms related to whitefly reproduction.

## Introduction

The whitefly *Bemisia tabaci* (Gennadius) is a species complex that contains some severe pests of vegetable, fiber, and ornamental crops [1], [ 2]. Two cryptic species of the *B*. *tabaci* complex, Middle East-Asia Minor 1 (MEAM1, formerly referred to as the “B biotype”) and Mediterranean (MED, formerly referred to as the “Q biotype”) have spread from their presumed origins in the Mediterranean regions into much of the world over the past 20 years, and have displaced some indigenous whitefly species in the regions of invasion [1]–[5]. The MEMA1 whitefly was first found in China in the late 1990s and is now the predominant or only species in many regions of the country [6]–[7]. However, the physiological mechanisms underlying this displacement are still unclear. Mating behavioral interactions between different species of whiteflies have been shown to contribute to the invasion success of MEAM1 [3], [ 8]. Individuals of MEAM1 feeding on begomovirus-infected plants may substantially increase their longevity and fecundity compared to those feeding on uninfected plants, while the indigenous ASIA II 3 whitefly (formerly referred to as “ZHJ1 biotype”) does not increase reproduction on virus-infected plants [9]. We verified that ovarian development in MEAM1 benefits from interaction with the viruses via the host plant [10] and it was proposed that such a vector-virus mutualism mediated through the host plant may accelerate the population increases of invasive whitefly in the field [9]. However, the physiological and molecular mechanisms underlying the increased reproduction of whitefly feeding on begomovirus-infected plants are not well understood.

Although viruses are most often studied as pathogens, many can be beneficial to their hosts, providing essential functions in some cases and conditionally beneficial functions in others [9], [ 11], [ 12]. Beneficial viruses have been discovered in many different hosts, including bacteria, insects, plants, fungi and animals [9], [ 13], [ 14]. How these beneficial interactions have evolved is poorly known. Our previous work showed that the MEAM1 whitefly benefits from its association with the tomato yellow leaf curl China virus (TYLCCNV) through their host plant by an increase in fecundity [9]. It was hypothesized that this benefit was due to enhanced synthesis and uptake of yolk protein [10]. Here, we test this hypothesis by characterizing the main yolk protein (Vitellin, Vt) and vitellogenesis in the MEAM1 whitefly.

Biochemical and molecular characterization of Vt and its precursor (vitellogenin, Vg) has been studied extensively in insects. These proteins provide the nutrition for egg development during oogenesis and embryonic development [15]. During vitellogenesis, yolk protein precursors are mostly produced in the fat body, secreted into the hemolymph and accumulated in the developing oocytes by a receptor-mediated way during oogenesis [16]–[18]. Vitellogenesis is a heterosynthetic process during which the synthesis of yolk resources is critical to egg maturation and the support of embryonic growth after oviposition [19], [ 20]. Understanding insect vitellogenesis is critical for elucidating the reproductive mechanism of insects and may provide knowledge for developing new strategies of insect pest control.

A few aspects of the morphological, biochemical, molecular, and biological characteristics related to oogenesis in *B*. *tabaci* have been studied previously. The *B*. *tabaci* female reproductive system consists of a pair of ovaries, each containing more than 12 ovarioles at different developmental stages [10], [ 21]. The morphology of reproductive organ in MEAM1 has also been described [22]. Oogenesis in MEAM1 initiated before adult emergence and virus-infected plants have indirect effect on whitefly reproduction [10]. However, our knowledge concerning the physiology and biochemistry of yolk proteins and vitellogenesis in whiteflies is limited, although some genomic information exists for whiteflies [23]–[25]. One reason is the difficulty of working with such a tiny insect, especially collecting hemolymph to monitor Vg dynamics during vitellogenesis [26]. Indirect double antibody sandwich ELISA on the basis of monoclonal antibodies against Vt, which can detect the level of ng of yolk protein, was successfully developed for monitoring vitellogenesis in several small insects [20], [ 27]–[30].

In this study we isolated and characterized Vt from eggs of MEAM1 of the *B. tabaci* species complex, developed monoclonal antibodies against Vt and cloned the Vg gene. We compared Vg gene expression level, hemolymph Vg and ovary Vt protein levels in individuals feeding on virus-infected vs. uninfected tobacco plants. To help separate the direct effects of the virus from indirect effects of the virus mediated via the plant on whitefly reproduction, we further examined the Vg mRNA levels and fecundity of whiteflies feeding on an artificial diet containing purified virions.

## Materials and Methods

### Whitefly Culture, Virus Inocula and Host Plants

The MEAM1 (mtCO1 GenBank accession no: GQ332577), MED (mtCO1 GenBank accession no: DQ473394) and Asia II3 (mtCO1 GenBank accession no: DQ309077) cryptic species of the whitefly *B*. *tabaci* species complex were reared on cotton (*Gossypium hirsutum* L.) cv. Zhemian 1973, a non-host plant of TYLCCNV [6], [9], [13], [31]. Infectious clones of TYLCCNV and their satellite DNA molecules (TYLCCNB) constructed previously [32], [ 33] were used as inocula for tobacco plants (*Nicotiana tabacum* L.) cv. NC89. To obtain virus-infected tobacco, plants at the 4–5 true-leaf stage were inoculated with either TYLCCNV (symptomless plants) or TYLCCNV+TYLCCNB (symptomatic plants) by agroinoculation as described previously [32]–[34]. Control plants were mock-inoculated with the *Agrobacterium tumefaciens* strain EHA105. All plants were watered every 3–4 days as necessary and fertilized once a week, and were grown to the 6–7 true-leaf stage for experiments (approximately 25 days after agroinoculation of the virus). The virus infection status of the test plants was judged by the characteristic symptoms and further confirmed by molecular markers as previously described [9]. All experiments were conducted at 26°C (±1°C), 40–60% RH and L: D 14 h: 10 h light regime.

### Artificial Feeding

Whiteflies were reared using an artificial diet system as previously described [35] with some modification. Up to 50 female adults were transferred from the cotton plants to a feeding chamber, a standing cylinder (glass tube), 35 mm in diameter and 50 mm in height, covered on top with a double layer of Parafilm (BEMIS, USA) with 200 µl of a diet containing 10% (w/v) sucrose and 1% (w/v) MES monohydrate. The whiteflies were reared in the chamber for one week in an incubator at 24°C, illuminated from above with a constant yellow light. The bottom was covered with a layer of gauze preventing escape of whitefly. Purified TYLCCNV (details of the methods are described in Information S1), inactivated TYLCCNV or equal volume of phosphate buffer (PBS) was added to the diet to set up three treatments. In each of the three treatments, three experimental replicates of adults feeding on the diets were sampled on 1d, 4d and 7d for Vg transcript level detection. Whether the virions have been transported into whitefly was further detected using PCR from the single DNA sample of each treatment.

### Ovary Sample Preparation

Adult females were anaesthetized on ice and ovaries were dissected by tearing the epidermis in a drop of cold PBS as previously described [10]. The ovaries were collected into a sterilized Eppendorf tube containing 100 µl PBS embedded in ice, homogenized quickly with a glass rod and centrifuged at 12,000 g for 15 min at 4°C. The supernatants were collected and stored at −70°C before being used to measure Vt uptake by the ovaries. Twenty-five pairs of ovaries were dissected each day, every 5 of them constituting one sample. A total of 25 pupae at the red eye stage, 250 adult females at different developmental stages were dissected.

### Hemolymph Sample Preparation

Hemolymph samples were prepared by dissecting adult females as described above without breaking the ovaries or other organs following the sampling method for tiny insects [20]. The resulting 50 µl of extract was then transferred to a sterilized Eppendorf tube containing another 50 µl of PBS on ice and centrifuged for 15 min at 12,000 g and 4°C to remove debris. The supernatants were collected and stored at −70°C for measuring Vg levels. The stages of the adult females were identical to those for the sampled ovaries.

### Purification of Vitellin

The egg samples (for method of sample preparation, see Information S1) were first purified by adding an equal volume of saturated ammonium sulfate solution, kept overnight at 4°C; subsequently, they were centrifuged at 10,000 g for 10 min, and the precipitate was dialyzed in cold PBS buffer (pH 7.4) for 24 h under constant stirring then diluted to 10 ml with buffer A (10 mM Tris-HCl, pH 7.8). The collected fractions were then applied to UNOTM Q1 ion exchange column (Bio-Rad) previously equilibrated in buffer A, and eluted with a linear NaCl in buffer A (0.0–1.0 M, pH 7.8). All procedures were run on a Bio-Logic Duo Flow TM system (Bio-Rad, USA). The purity of Vt was verified by SDS – PAGE and Western, and the concentration of the purified Vt was estimated with protein assay kits (Bio-Rad, USA). The purified Vt was used as ELISA standard and for preparing antibodies.

### Western Blot and Gel Electrophoresis

To detect the yolk protein in individuals of MEAM1 feeding on cotton, whole bodies of 100 whiteflies were collected into sterilized Eppendorf tubes containing 30 µl PBS embedded in ice, homogenized with a glass rod and centrifuged at 12,000 g for 15 min at 4°C. The supernatants were collected and stored at −70°C before being used. Methods are provided in detail in Information S1.

### Immunocytochemistry

Oocytes of MEAM1 adult females at various development phases were acquired using the method previously described [10]. The ovaries were first dissected in 0.02 M phosphate-buffered saline (PBS) at pH 7.4, and ovarioles were isolated individually. The isolated ovarioles were fixed in 4% formaldehyde (freshly prepared from paraformaldehyde) in PBS for 30 min at room temperature and then were extracted with 0.1% Triton X-100 for 30 min. After rinsing in PBS, the follicles were blocked with 1% with bovine serum albumin (BSA) for 1 h at room temperature and then incubated overnight at 4°C with a mouse anti-Vt monoclonal antibody (10 mg/mL in PBS) (see Information S1). Subsequently, the ovarioles were thoroughly washed in PBS and incubated for 1 h at room temperature with a goat anti-mouse antibody conjugated to FITC (Sigma Chemical, Heidelberg, Germany; dilution 1∶100). The follicles were then stained with DAPI (40-6-diamidino-2-phenylindole) (Sigma; 1 µg/mL in PBS) for 20 min at room temperature. The labeled specimens were washed three times in PBS. Samples were observed and photographed using a Nikon C1 Confocal Microscope Imaging System (Nikon, Japan).

### ELISA

The method of detecting Vg/Vt in hemolymph or ovary was as described by Dong et al. [20] with following modifications. Polyvinyl 96-well microplates (NUNC, Denmark), coated with purified polyclonal antibody, were washed three times with TBST (20 mM Tris, pH 7.5; 150 mM NaCl; 0.05% Tween-20). After incubating with 1% bovine serum albumin (BSA), the hemolymph and ovarian samples (100 µl/well) were added into the coated plates and incubated for 90 min followed by another triple washing with TBST. A dilution series of purified MEAM1 whitefly Vt as the reference standard was used. Then, the purified mouse antiserum against whiteflies Vt and the Horseradish Peroxidase (HRP)-labeled goat anti-mouse conjugate (Sigma) (1∶10,000 dilution) was added separately to each well (100 µl/well) and incubated for 60 min. The enzyme substrate 3, 3′, 5, 5′-Tetramethylbenzidine (Sigma) was added after washing with TBST for three times and another TBS for 5 min. The colorimetric readings were recorded 30 min later with a BIO-TEK® ELISA reader at OD_405nm_.

### Expression and Analysis of Vg mRNA Level

Twenty-five days after inoculation of tobacco plants, whiteflies in three replicate plants for each treatment were sampled. For analysis of Vg mRNA level, fat bodies were dissected from female adult whiteflies of different stages fed on uninfected and infected host plant which were inoculated with EHA105, TYLCCNV or TYLCCNV+TYLCCNB. Samples fed on artificial diet containing purified TYLCCNV, inactivated TYLCCNV or only phosphate buffer were acquired in the same way. Briefly, at least 100 female adult whiteflies were dissected in a drop of cold DEPC buffer and fat bodies were collected into RNAse free tube for isolating total RNA. RNA extraction and cDNA synthesis were conducted using the methods described in Information S1. Primers for target Vg sequence and endogenous control *β*-actin are as follows: Vg sense- ACAAGTCTCCGACGCCGAAG, Vg antisense-TTGACATCGGCTTTACGGCA; actin sense- TCACCACCACAGCTGAGAGA, actin antisese- CTCGTGGATACCGCAAGATT. The amplification conditions used were 3 min at 95°C, followed by 40 cycles of 30 s at 95°C, 30 s at 56°C, and 30 s at 72°C. Expression levels were detected using a Real-time quantitative PCR performed on ABI 7500 (Applied Biosystems, USA). The relative expression levels were calculated according to the ΔΔCT method [36], [ 37].

### Fecundity of Female Adults

The number of eggs laid was examined following the previous method [10] using leaf clip-cages. One week after they have fed on artificial diets or tobacco plants, female adults were transferred to uninfected tobacco plants covered by clip-cages. Ten pairs of adults of each treatment were used. Each cage contained one male and one female. The total number of eggs laid was examined using a microscope (Leica, Germany) one week after transfer to uninfected plants.

### Data Analysis

All statistical analysis were conducted using the DPS^©^ package (Version 10.01 for Windows) [38]. The effects of virus infection and age on the Vg and Vt levels were analyzed using two-factor ANOVA and Tukey’s multiple range test. To compare the difference in the levels of Vg and Vt of whiteflies feeding on healthy and virus-infected plants, data of Vg mRNA levels in TYLCCNV-infected and TYLCCNV+TYLCCNB co-infection plants, data in whiteflies feeding on purified TYLCCNV and inactive TYLCCNV were subjected to Student’s *t*-test. Data of egg laid was analyzed in the same way. All tests were considered significant at *P*<0.05.

## Results

### Characterization of Vitellogenin and Vitellin in Whiteflies

**Figure 1:**
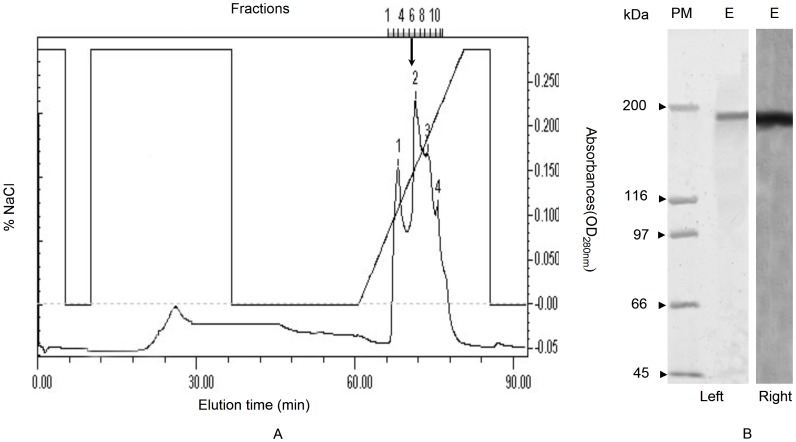
Elution profile of crude vitellin from UNOTMQ1 ion exchange column. Vitellin was eluted with a gradient of 0.0–1.0 M NaCl in Tris–HCl buffer pH 7.8 (A); fractions indicated with arrow were pooled as purified Vt, verified using SDS–PAGE (B, left) and Western blot (B, right) with monoclonal antibodies against *B*. *tabaci* Vt. E: purified Vt; PM: prestained molecular weight standards.

A specific protein band (∼380 kDa) can only be detected in adult females, which was stained positive for carbohydrate, lipid, and phosphorus, thus demonstrating that this protein was a Vt in whiteflies (Fig. S1). Vt in the ovary of MEAM1 was purified by the combined use of gel filtration and ion-exchange chromatography (Fig. 1A). Purified Vt was composed of two subunits with approximate molecular weights of 190 kDa (Fig. 1B). Based on the purified Vt, monoclonal antibodies were developed (Fig. S2). These antibodies reacted not only with Vg/Vt from MEAM1 (Fig. S3), but also with those from two other whitefly species, MED and ASIA II3 (Fig. S4). The monoclonal antibodies showed higher specificity to binding soluble proteins from adult female hemolymph, ovary and whole body extracts in MEAM1 females, but had no binding to those protein from adult males (Fig. S3).

Vg gene was sequenced and found to be 6474 bp, encoding 2158 amino acids (Genebank No.: GU332720). The deduced amino acid sequence demonstrated that the molecular weight of Vg subunit was approximately 190 kDa (Fig. S5). A phylogenetic analysis of amino acid sequences (without signal peptide) from MEAM1 Vg and 32 Vgs derived from 26 insect species revealed that the tree clustered *B*. *tabaci* and other insects in Hemiptera into a specific order (Hemiptera) in a coherent manner (Fig. S6).

### Vitellogenesis of MEAM1 Whitefly

**Figure 2:**
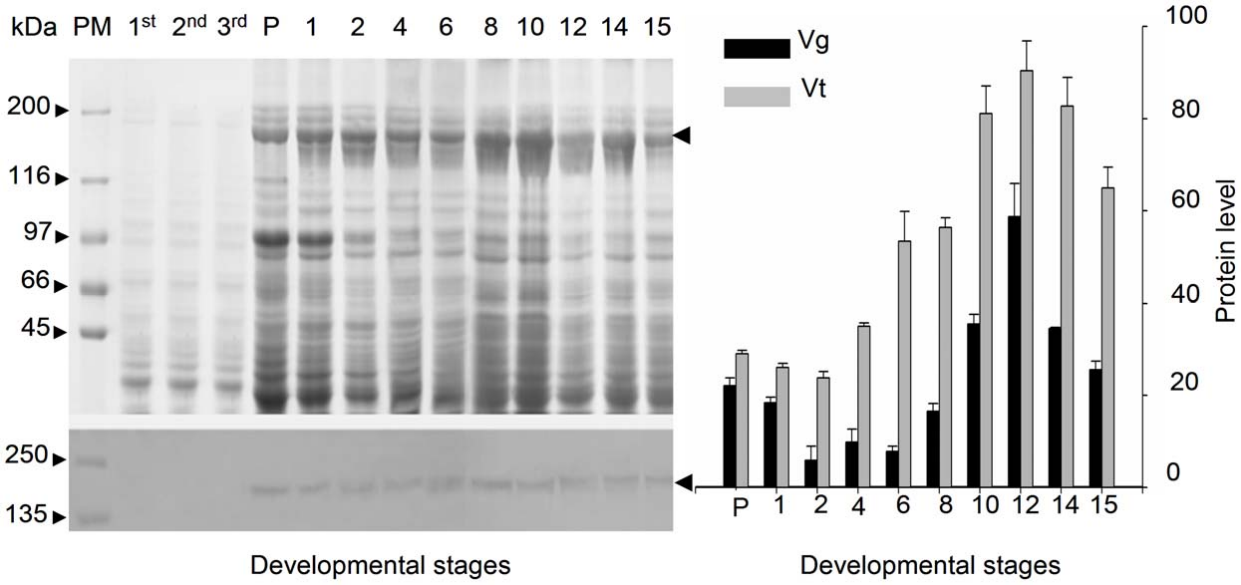
Vitellin and vitellogenin levels in MEAM1 whiteflies fed on cotton. Yolk soluble protein was examined using SDS – PAGE (left above) and corresponding Western blotting analysis (left bottom). Levels of hemolymph Vg (black column) and ovary Vt (gray column) in MEAM1 whitefly at different developmental stages were monitored using sandwich ELISA (ng/female). Arrows indicate Vg or Vt. PM: prestained molecular weight standards; P: red eye pupae; 1^st^, 2^nd^ and 3^rd^: the first, second and third instars nymph; 1–15: days after eclosion. Data: Means±S.E. (*n* = 25).

A sandwich ELISA was developed using purified monoclonal antibodies (Fig. S3) and polyclonal antiserum made against MEAM1 Vt. Both Vg and Vt levels in adult females fed on cotton plants were detected using Western blot and sandwich ELISA (Fig. 2). Vg initiated expression at red eye pupal stage (Fig. 2 left). And then, there is a slight increase during the first 6 d after eclosion (Fig. 2 right). After this stage, synthesis of Vg increased rapidly until 12 d and then decreased until death. The highest Vg peak of 60 ng per female was observed at 12 d after eclosion (Fig. 2 right). Similar dynamics of Vt levels was observed in the ovaries, and the highest Vt level in the ovary was 90 ng per female (Fig. 2 right).

**Figure 3:**
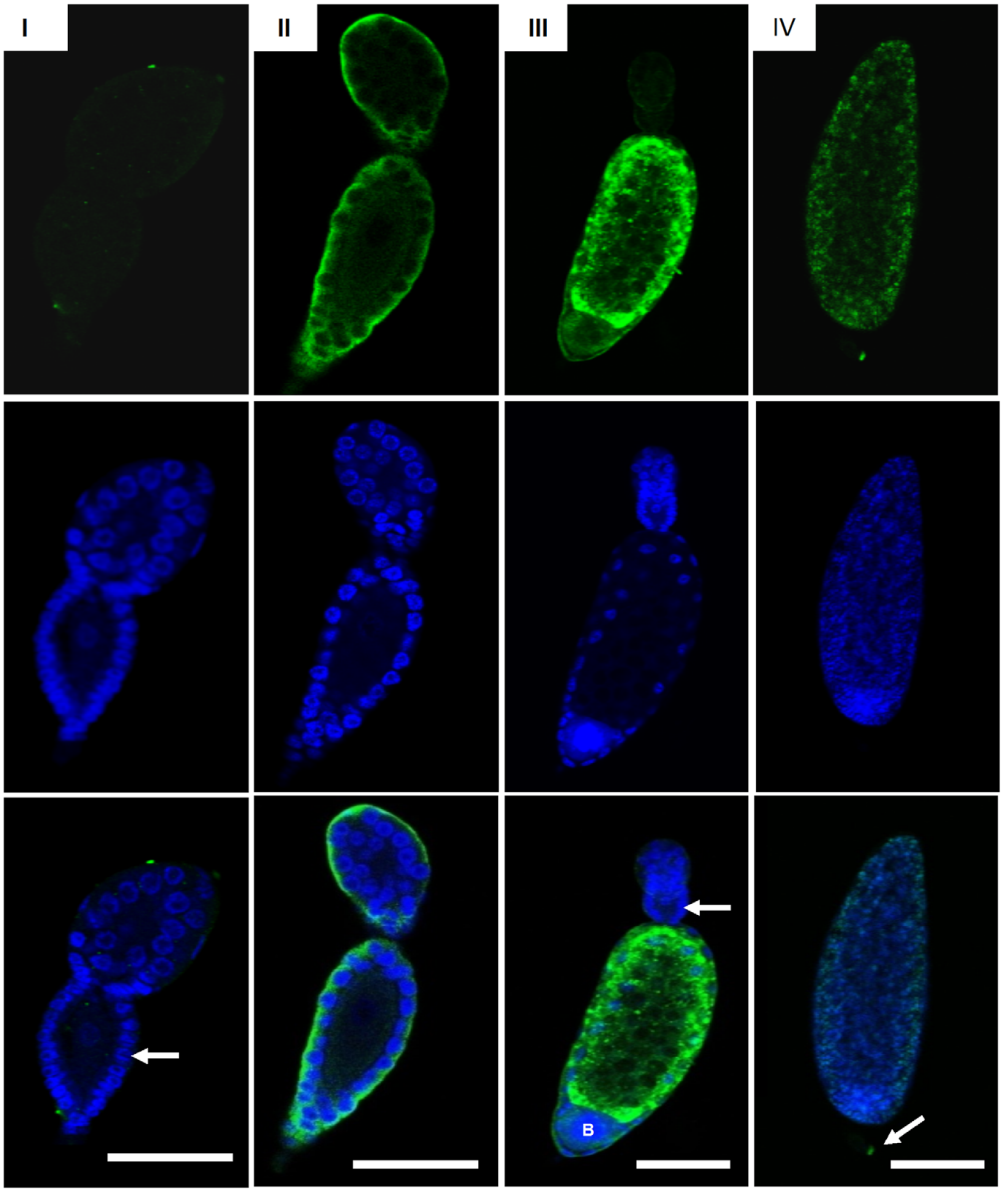
Localization of vitellin in different phases of ovarioles during vitellogenesis of MEAM1 whiteflies. The nuclear and vitellin were seen by laser scanning confocal microscopy following exposure to DAPI (blue) and FITC labeled anti-Vt antibody (green). I: previtellogenesis-phase I oocytes. More than 20 follicular cells (white arrow) surround the oocyte was observed. No signal of vitellin was detected. II: early vitellogenesis-phase II oocytes. Vitellin (white arrow) was observed around the follicular cell. III: mid vitellogenesis-phase III oocytes. Vitellin (white arrow) was formed spheres and filled most space of the oocyte. At the top of oocytes, a previtellogenesis oocyte was formed. A bacteriocyte sphere (showed with ‘B’) was uptake by the developmental oocyte. IV: post vitellogenesis-phase IV oocytes. Vitellin has filled with oocytes and formed mature oocytes. Egg pedicel (white arrow) was observed. The details of different phase of oocytes were described in Guo et al. [9]. Bars = 50 µm.

From analysis of immunocytochemistry, Vt was first detected in phase II oocyte, and Vt mainly existed around the follicular cell in this stage (Fig. 3). The phase II oocyte first occurred in the ovaries of red eye pupal stage whiteflies, suggesting that vitellogenesis was initiated during this stage, which is in accord with the result of Western blot analysis (Fig. 2). The phase III oocyte was filled with Vt, revealing that Vt was rapidly absorbed during this stage. A bacteriocyte sphere was obviously observed in the phase III oocyte, which stained strongly positive to DAPI. The phase III oocyte was mainly in the ovaries of whiteflies 8–14 d after eclosion, suggesting that these whiteflies were in vitellogenesis. The vitellin membrane formed in the phase IV oocyte, and the oocytes could not be well stained by anti-Vt antibody because the vitellin membrane retarded antibodies permeating into the follicle (Fig. 3).

### Increased Vitellogenin and Vitellin Levels in MEAM1 Feeding on Virus-infected Plants

**Figure 4:**
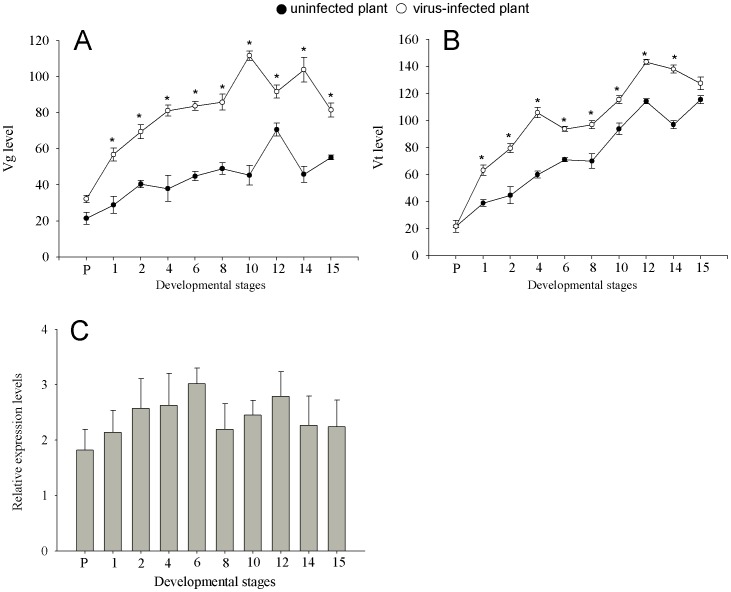
Comparison of vitellin and vitellogenin levels in whiteflies feeding on uninfected and virus-infected plants. Virus-infected plants were inoculated with TYLCCNV+TYLCCNB. Expression patterns of hemolymph Vg (A) and ovarian Vt (B) (ng/female): all the data are expressed as Mean±S.E. (*n* = 25). On the same day, values followed by the asterisk (*) represent significant differences (*P*<0.05, Student *t*-test). Fat body Vg mRNA (C): Grey columns correspond to Vg mRNA transcript levels using *β*-actin as reference according to the ΔΔCT method. Mean±S.E. of three biological and three experimental replicates are represented.

MEAM1 showed an increased synthesis of Vg and Vt after eclosion while feeding on either uninfected or virus-infected (inoculated with TYLCCNV+TYLCCNB) tobacco plants. However, compared to those of the females fed on uninfected tobacco plants, Vg and Vt levels were significantly higher in females fed on virus-infected plants (Table 1, Fig. 4). The highest level of Vg in the hemolymph was approximately 110 ng per female (Fig. 4A) and highest Vt level in the ovary was approximately 140 ng per female (Fig. 4B) when fed on virus-infected plants. The relative expression level of Vg mRNA was increased after eclosion. Compared to those feeding on uninfected plants, the Vg mRNA levels were higher than that in whiteflies feeding on virus-infected plants, the highest Vg mRNA relative expression level was three times higher observed at 6 d after eclosion (Fig. 4C).

**Table 1:** Two-factorial ANOVA on yolk protein level in MEAM1 whitefly feeding on uninfected and virus-infected plants.

Parameters measured	Protein level (ng/female)		Two-factors ANOVA
	Uninfected	Virus-Infected	
Vitellogenin	102±1.734a	121±1.444b	*F*_A_ = 5.194, *df* = 1; *P*<0.05
			*F*_B_ = 41.982, *df* = 12; *P*<0.05
			*F*_A×B_ = 5.374, *df* = 12; *P*<0.05
Vitellin	98±1.896a	123±1.819b	*F*_A_ = 5.797, *df* = 1; *P*<0.05
			*F*_B_ = 34.944, *df* = 12; *P*<0.05
			*F*_A×B_ = 3.683, *df* = 12; *P*<0.05

Note: the values for each parameter measured are from all data during the whole experiment period, and are expressed as mean±standard error (*n* = 250). Virus-infected plants were inoculated with TYLCCNV+TYLCCNB. In the same row, means followed by different lowercase letters differ significantly according to two-factors ANOVA. Factor A: types of tobacco plants for whitefly (Virus-infected plants versus uninfected plants); Factor B: the time of different developmental stages.

### Co-infection of Plants by TYLCCNV+TYLCCNB Increased Whitefly Vg Gene Transcript Level and Fecundity

**Figure 5:**
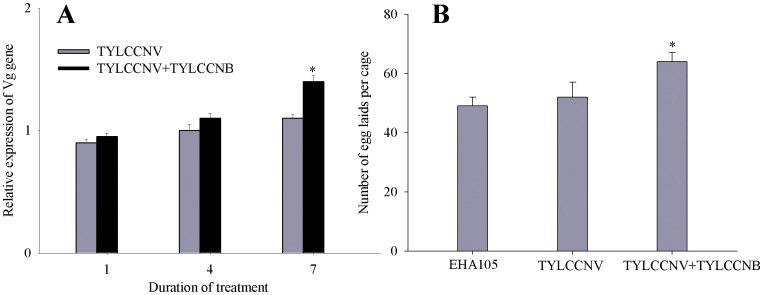
Effect of TYLCCNV+TYLCCNB co-infection of plants on the fat body Vg mRNA levels and fecundity in whiteflies. Relative transcript levels of Vg gene (A) and realized fecundity (B) in EHA105-infiltrated, TYLCCNV-infected and TYLCCNV+TYLCCNB co-infected plants were compared. Mean±S.E. of three biological and three experimental replicates are represented. Values followed by an asterisk (*) represent significant differences (*P*<0.05, Student *t*-test).

**Figure 6:**
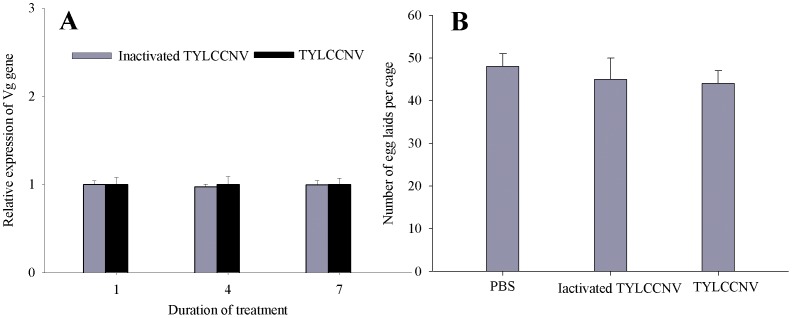
Effects of artificial feeding with purified TYLCCNV on the Vg mRNA levels and fecundity in whiteflies. Relative transcript levels of Vg gene (A) and realized fecundity (B) of adults feeding on diets containing purified TYLCCNV, inactivated TYLCCNV and equal volume of phosphate buffer (PBS) were compared. Mean±S.E. of three biological and three experimental replicates are represented. Values followed by an asterisk (*) represent significant differences (*P*<0.05, Student *t*-test).

To find out whether TYLCCNV itself influences the vitellogenesis, we compared the transcription of Vg gene in *A*. *tumefaciens* EHA105-infiltrated, TYLCCNV-infected and TYLCCNV+TYLCCNB-co-infected tobacco plants. The transcript level for the Vg gene was increased in whiteflies feeding on TYLCCNV+TYLCCNB co-infected plants when compared to that feeding on TYLCCNV-infected plants (Fig. 5), and in addition, did not change between whiteflies feeding on artificial diets containing either purified TYLCCNV or inactivated TYLCCNV (Fig. 6). Moreover, significantly higher numbers of eggs were laid by whiteflies feeding on TYLCCNV+TYLCCNB co-infected plants when compared to whiteflies feeding on TYLCCNV-infected plants (Fig. 5), while whiteflies feeding on artificial diets containing purified TYLCCNV or inactivated TYLCCNV had similar numbers of eggs laid (Fig. 6).

## Discussion

In most insects, Vg has two or more subunits, usually with a big subunit (150–200 kDa) and a smaller one (50–60 kDa), while some insects have only large subunits (185–200 kDa) [18], [ 19]. The yolk protein of the higher Diptera is an exception, since it consists of three to five polypeptides of 44–51 kDa [16], [ 39]. In *B*. *tabaci*, the molecular mass of native Vg is appropriately 380 kDa and is composed of two subunits with approximate molecular weights of 190 kDa, which is similar to those insects with only large Vg subunits, such as parasitic wasps, *Pimpla nipponica* [40] and *Pteromalus puparum* [20]. In addition, Vgs and Vts share immunological properties and are physically and chemically similar in most insects [16]. In *B*. *tabaci,* Vg and Vt are also immunologically identical, anti-Vt monoclonal antibody can recognize Vg in the hemolymph, Vt in the ovaries and egg extracts of reproductive females, and also react with those proteins from ASIA II3 and MED whiteflies (Fig. S4). It was assumed that similar epitopes occurred in the yolk proteins from different species of whiteflies. Therefore, this monoclonal antibody can be used as a probe to detect the dynamics of Vg and Vt levels in other whiteflies. A comparison of vitellogenesis of MEAM1 whitefly with other species may help to reveal the mechanisms of whitefly species replacement as a result of difference in fecundity.

During vitellogenesis, Vg is produced mostly in female fat body, secreted into the hemolymph and accumulated in the developing oocytes by a receptor-mediated way during oogenesis [16], [18]. In MEAM1, vitellogenesis initiated at red eye pupal stage and consisted with the high Vg mRNA expression level during this stage, and the highest levels of Vg mRNA was observed at 6 d after eclosion. However, the highest levels of hemolymph Vg and ovary Vt were observed at 12 d after eclosion. This is similar to the sawfly, *Athalia rosae ruficornis*, in which Vg production began in the late pupal stage [41]. However, in two other hemipterous insects including the bean bug, *Riptortus clavatus* [42] and the rice planthooper, *Nilaparvata lugens* [43], Vg initiated synthesis 2–3 days after eclosion and reached a maximum on day 7.

It has been shown that the invasive MEAM1 whitefly feeding on begomovirus-infected plants substantially increases its longevity and fecundity compared to that on uninfected plants while the indigenous ASIA II3 whitefly is unable to do so [9]. Previously we demonstrated that MEAM1 benefits from the indirect virus mutualism in some manner associated with ovarian development [10] and this benefit was assumed due to the enhanced synthesis and uptake of yolk protein. In this study, we show that levels of hemolymph Vg and ovary Vt in the females fed on virus-infected tobacco plants were significantly higher than those in females fed on uninfected tobacco plants. A significant higher expression of Vg mRNA level in whiteflies fed on virus-infected plants was also observed. These data together reveal that MEAM1 individuals fed on virus-infected plants improved the levels of Vg synthesis and uptake.

Vitellogenesis can be affected by many factors, including hormone, stress and nutrition [44], [ 45], among others. In this study, we demonstrated that vitellogenesis in MEAM1 whitefly can be indirectly enhanced through increasing Vg synthesis by a begomovirus via their shared host plant. This is further demonstrated by an increased transcript level of Vg gene in whiteflies feeding on TYLCCNV+TYLCCNB co-infected plants when compared with that of whiteflies feeding on either EHA105-infiltrated or TYLCCNV-infected plants. To our knowledge, this is the first report that a virus can accelerate vitellogenesis of its vector insect and thereby increase its fecundity via their shared host plant. In addition, it seemed that the indirect virus mutualism has effects only on the synthesis and uptake of Vg but not the timing of vitellogenesis in whiteflies. However, how begomoviruses act on the host plant to have effects on vitellogenesis of its vector insects is not yet completely understood. It has been reported that begomoviruses can directly increase the expression of genes involved in the cellular and humoral immune response in viruliferous MEAM1, but Vg gene expression in the viruliferous individuals is not changed following transfer to a non-host plant [25]. This result suggests that the virus itself does not directly affect the host insect Vg gene expression, but other changes in the host plant following virus-infection may contribute to the Vg gene expression. This is further demonstrated by the similar Vg gene transcript levels between whiteflies feeding on artificial diets containing purified TYLCCNV, inactivated TYLCCNV or only phosphate buffer (Fig. 6). Research on luteoviruses that infect wheat and potatoes and the respective aphid vectors demonstrated that virus infection of host plants enhances the life history of vectors [46], [ 47]. Additionally, it has shown that virus infection alters the concentration and relative composition of volatile organic compounds in host plants [48], [ 49].

Using the same whitefly-virus-plant system as that of this study, our recent work showed that virus-infection suppresses jasmonic acid-related defenses in the tobacco plants and impairing or enhancing defenses mediated by jasmonic acid in the plant enhances or depresses the performance of the whitefly [50]. Further, we showed that virus-infection of the tobacco plants does not seem to change the nutritional quality of phloem sap but whiteflies fed on virus-infected plants improve their nutritional assimilation [51]. The combined data of this and the two recent studies [50], [ 51] seem to indicate that a begomovirus may repress a plant’s defense to a whitefly, and the reduced plant defense in turn may allow the whitefly to elevate its primary metabolism, with the outcome of better nutritional assimilation and enhanced vitellogenesis. However, what defensive compounds are repressed in the virus-infected tobacco plants are yet to be identified. In view of the diversity of compounds in tobacco plants, definite identification of the key compounds responsible for the beneficial effects to the whitefly may present a great challenge. Nevertheless, our findings so far not only provide insights into the molecular and physiological mechanisms underlying the increased reproduction of a whitefly species feeding on a virus-infected plant, but also provide a better understanding of the molecular mechanisms that regulate whitefly reproduction.

## Supporting Information

Figure S1 **Characterization of MEAM1 whitefly Vt.** Native-PAGE (linear gradient consisting of 4–25% polyacrylamide) analysis with Coomassie brilliant blue staining (left) and the characterization of yolk soluble protein (right), Vt bands were visualized by staining with Coomassie brilliant blue (C), Sudan Black B (L), Periodic acid-Schiff’s reagent (P) and Methyl Green Solution (G). The soluble proteins sampled from eggs (E), ovaries (O), female (F) and male (M) adults 6 d after eclosion. PM: high molecular weight standards (Amersham). Arrows indicate the bands of Vg or Vt.
(DOC)

Figure S2 **Purification of monoclonal antibody against MEAM1 whitefly Vt.** SDS–PAGE of the purified monoclonal antibody IgG against *B*. *tabaci* Vt with Coomassie brilliant blue staining. PM: Molecular weight standards; A: purified IgG; B: crude ascites fluid; H and L: IgG heavy and light chain.
(DOC)

Figure S3 **Distribution of Vg/Vt in different tissues of MEAM1 whitefly.** SDS – PAGE analysis with Coomassie brilliant blue staining (left) and corresponding Western blotting analysis (right) with the monoclonal antibody against *B*. *tabaci* Vt for soluble proteins sampled from different tissues of the female and male. PM: prestained molecular mass markers (Bio-Rad); E: egg extract; H and O: female hemolymph and ovaries 6 d after eclosion; F and M: soluble protein of female and male adults 6 d after eclosion; Arrow indicates subunits of Vg or Vt.
(DOC)

Figure S4 **Immune reaction of Vt antibody with yolk protein of MED and ASIA II3 whiteflies.** SDS–PAGE analysis with Coomassie brilliant blue staining (left) and corresponding Western blotting analysis (right) with the monoclonal antibody against *B*. *tabaci* Vt for soluble proteins sampled from MEAM1, MED and ASIA II3 whiteflies. PM: prestained molecular mass markers (Bio-Rad); BF, QF and ZF: soluble protein of MEAM1, MED and ASIA II3 female adults 6 d after eclosion. BM, QM and ZM: soluble protein of MEAM1, MED and ASIA II3 male adults 6 d after eclosion; Arrow indicates subunits of Vg or Vt.
(DOC)

Figure S5
**Nucleotide and deduced amino acid sequence of the vitellogenin cDNA of MEAM1 whitefly,** ***Bemisia tabaci*** **.** “
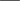
” signal peptide, “
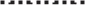
” functional motif, “
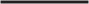
” polyserines, “
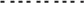
” unknown motif.(DOC)

Figure S6 **Phylogenetic tree of Vg in MEAM1** ***Bemisia tabaci*** **and other insects of their predicted amino acid sequences using the neighbor-joining method.**
(DOC)

Information S1 **Details of methods, sequences of vitellogenin cDNA.**
(DOC)
