# Supplementary figures and images for: Enhanced Vitellogenesis in a Whitefly via Feeding on a Begomovirus-Infected Plant
Source: PLoS One. 2012 Aug 24;7(8):e43567. doi: 10.1371/journal.pone.0043567 (PMC3427354; doi:10.1371/journal.pone.0043567)

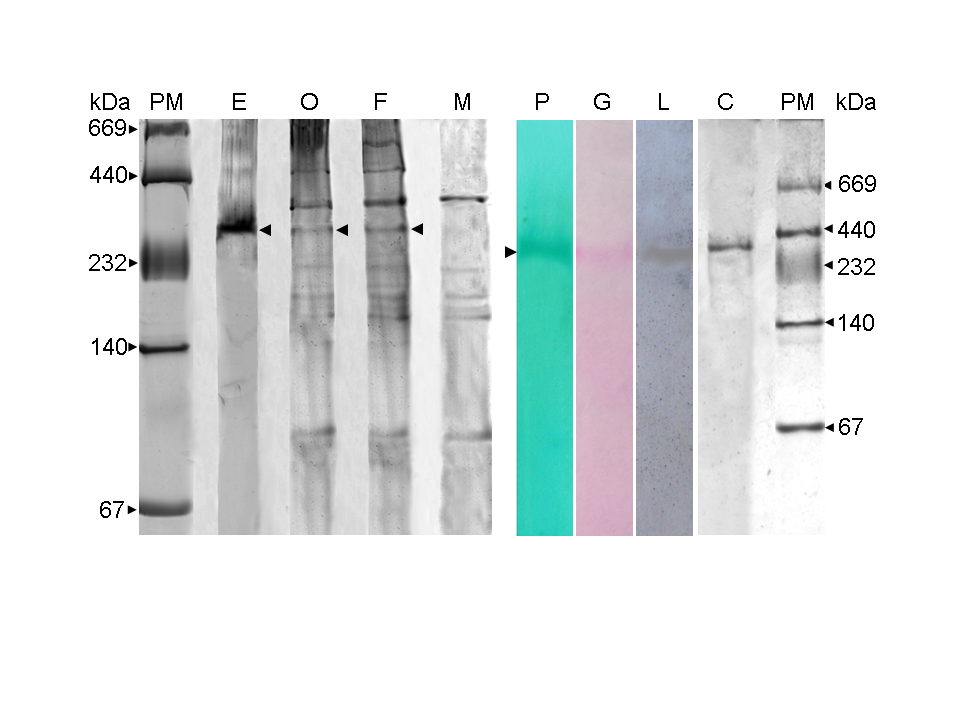

Supplement: Figure S1 — Characterization of MEAM1 whitefly Vt. Native-PAGE (linear gradient consisting of 4–25% polyacrylamide) analysis with Coomassie brilliant blue staining (left) and the characterization of yolk soluble protein (right), Vt bands were visualized by staining with Coomassie brilliant blue (C), Sudan Black B (L), Periodic acid-Schiff’s reagent (P) and Methyl Green Solution (G). The soluble proteins sampled from eggs (E), ovaries (O), female (F) and male (M) adults 6 d after eclosion. PM: high molecular weight standards (Amersham). Arrows indicate the bands of Vg or Vt. (DOC) [file pone.0043567.s001.doc]

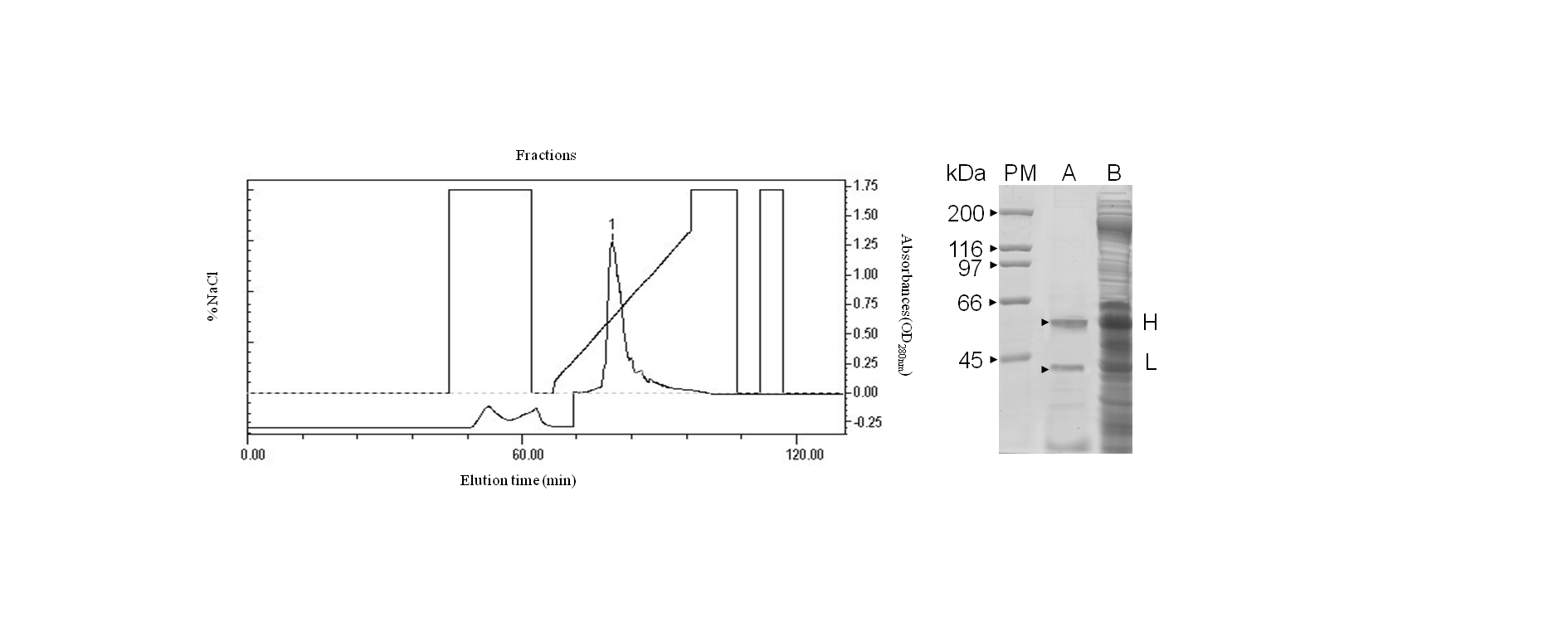

Supplement: Figure S2 — Purification of monoclonal antibody against MEAM1 whitefly Vt. SDS–PAGE of the purified monoclonal antibody IgG against B. tabaci Vt with Coomassie brilliant blue staining. PM: Molecular weight standards; A: purified IgG; B: crude ascites fluid; H and L: IgG heavy and light chain. (DOC) [file pone.0043567.s002.doc]

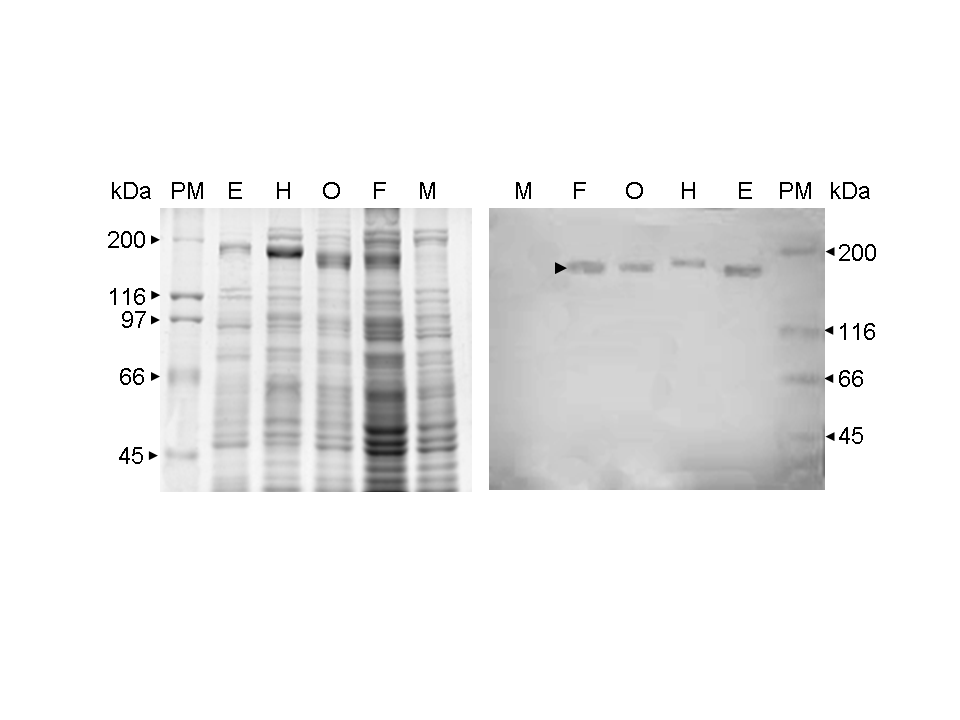

Supplement: Figure S3 — Distribution of Vg/Vt in different tissues of MEAM1 whitefly. SDS – PAGE analysis with Coomassie brilliant blue staining (left) and corresponding Western blotting analysis (right) with the monoclonal antibody against B. tabaci Vt for soluble proteins sampled from different tissues of the female and male. PM: prestained molecular mass markers (Bio-Rad); E: egg extract; H and O: female hemolymph and ovaries 6 d after eclosion; F and M: soluble protein of female and male adults 6 d after eclosion; Arrow indicates subunits of Vg or Vt. (DOC) [file pone.0043567.s003.doc]

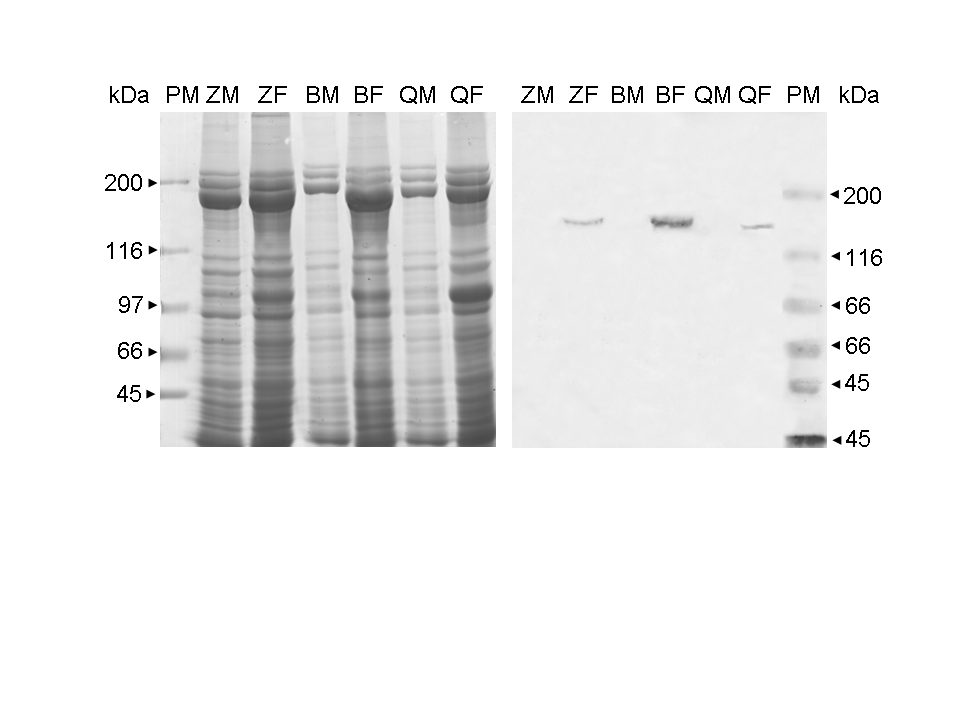

Supplement: Figure S4 — Immune reaction of Vt antibody with yolk protein of MED and ASIA II3 whiteflies. SDS–PAGE analysis with Coomassie brilliant blue staining (left) and corresponding Western blotting analysis (right) with the monoclonal antibody against B. tabaci Vt for soluble proteins sampled from MEAM1, MED and ASIA II3 whiteflies. PM: prestained molecular mass markers (Bio-Rad); BF, QF and ZF: soluble protein of MEAM1, MED and ASIA II3 female adults 6 d after eclosion. BM, QM and ZM: soluble protein of MEAM1, MED and ASIA II3 male adults 6 d after eclosion; Arrow indicates subunits of Vg or Vt. (DOC) [file pone.0043567.s004.doc]

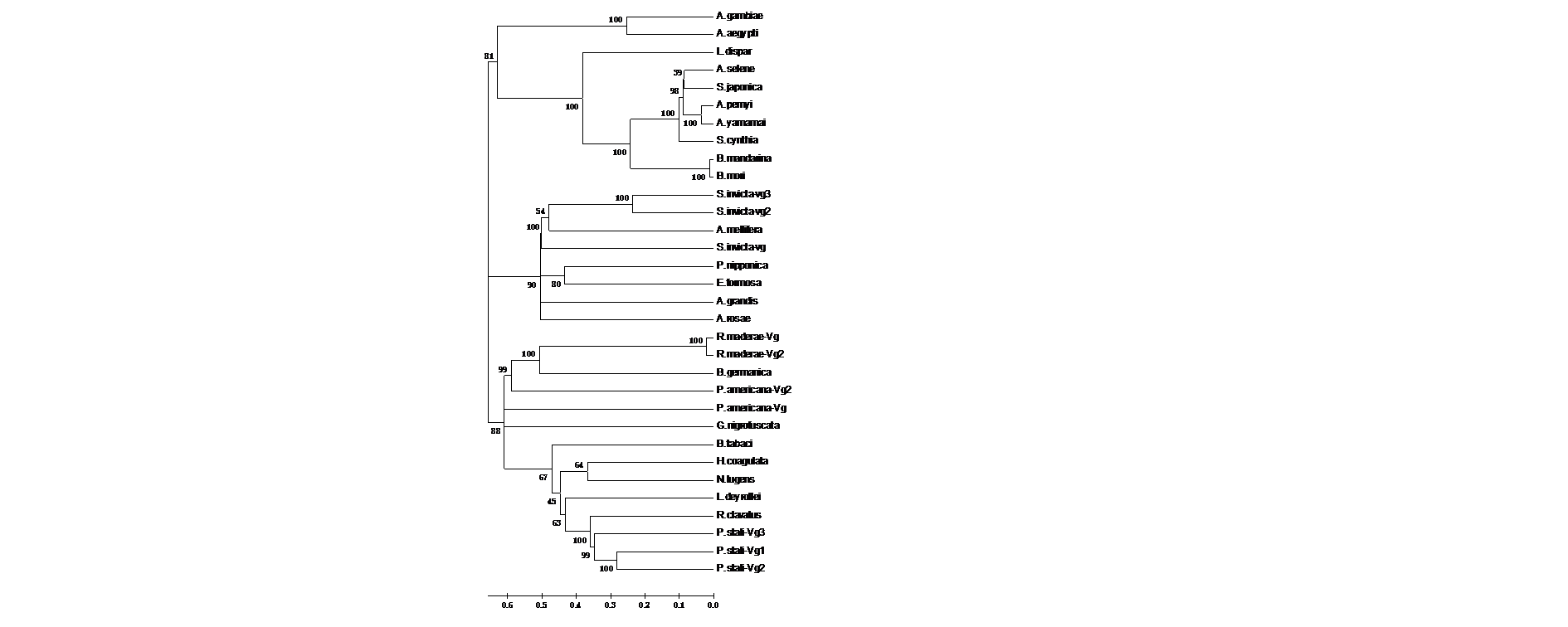

Supplement: Figure S6 — Phylogenetic tree of Vg in MEAM1 Bemisia tabaci and other insects of their predicted amino acid sequences using the neighbor-joining method. (DOC) [file pone.0043567.s006.doc]
