# Supplementary material for: Enhanced Vitellogenesis in a Whitefly via Feeding on a Begomovirus-Infected Plant
Source: PLoS One. 2012 Aug 24;7(8):e43567. doi: 10.1371/journal.pone.0043567 (PMC3427354; doi:10.1371/journal.pone.0043567)
Supplement: Figure S5 — Nucleotide and deduced amino acid sequence of the vitellogenin cDNA of MEAM1 whitefly, Bemisia tabaci . “” signal peptide, “” functional motif, “” polyserines, “” unknown motif. (DOC) [file pone.0043567.s005.doc]

Translation of B Vg-ORF(1-6474)

Total amino acid number: 2157, MW=242561

1 ATGTGGACTCCCGTATTATTGTGCCTGCTGGTCGCAGCCGCCAATGCGCAATATGGATGG

1 M W T P V L L C L L V A A A N A Q Y G W

61 AAAAATGGAAACTTGTACAAATATGAAGTCAACGGACGCACTCTGACCGCCCTCAACCAA

21 K N G N L Y K Y E V N G R T L T A L N Q

121 GTTGCCGACCAATATGCCGGAGTCCTATTCAGAGCCAACTTCATCGTCCAACCTCTCTCC

41 V A D Q Y A G V L F R A N F I V Q P L S

181 AGTGACAGATTATCCGCCCAGATCCAAAATGCCGAAATCGCTCAAGTTCACACTGAACTC

61 S D R L S A Q I Q N A E I A Q V H T E L

241 CCTGCTGGTTATGAATCTCACATTCCCTCCAGTCAGTTGAATTACAAGAACATGCCCCTC

81 P A G Y E S H I P S S Q L N Y K N M P L

301 AGCGAACAACCATTCGAAATTAACCTGAAGCAAGGAGTTGTCTCCAACCTTCGCGTTAAC

101 S E Q P F E I N L K Q G V V S N L R V N

361 AAGAATGTCTCCGACTGGGAACTCAACATCATCAAGGCTGTTGTGAGCCAAATCCAAGTT

121 K N V S D W E L N I I K A V V S Q I Q V

421 GACACCCAAGGTCAAAACTTGAAGAAATCCAGCCACAACCAACTCCCCAAGGAAAACAAG

141 D T Q G Q N L K K S S H N Q L P K E N K

481 CCCTACGGTGTTTACAAGACCATGGAAGACTCCGTCACCGGTGAATGTGAAACTCTCTAC

161 P Y G V Y K T M E D S V T G E C E T L Y

541 GATGTCTCACCTCTGCCAGAGATTACCCTCCAAACTAGACCCTGGTTGGTTCCTTTCCCC

181 D V S P L P E I T L Q T R P W L V P F P

601 AAGCTCCGTGAAAACGGACAAGTCATCGACATCGTCAAGGCCACCAACTACAGCAAATGT

201 K L R E N G Q V I D I V K A T N Y S K C

661 GAAGAACGTTCTGCCTACCACTTTGGTATCACTGGTCTCACCAACTGGAAACCCGCCAGC

221 E E R S A Y H F G I T G L T N W K P A S

721 AACCAGATGGGACAGTTCCTCTCCCGCTCCAACATCAACCGTGTCATTATCTCCGGCAAT

241 N Q M G Q F L S R S N I N R V I I S G N

781 GTGAAGCAATACCACATCCAATCCTCTGTCTCTACCAACAAAATCGTCATCAGCCCACAG

261 V K Q Y H I Q S S V S T N K I V I S P Q

841 ATGTACGAATCACAGAAGGGAATGGTTGTCAGTGTCATGAACTTGRCTTTGGCCTCCTTC

281 M Y E S Q K G M V V S V M N L X L A S F

901 CACCAATCCAACGGATCTCCTCGTATGGTCTCCAACGCCCGCAAGATCAACAACTTGGTC

301 H Q S N G S P R M V S N A R K I N N L V

961 TACGACTACAACGCTGCCTCCCCAAACGCATACGCTCAACACTACAACAACAATGGTGCA

321 Y D Y N A A S P N A Y A Q H Y N N N G A

1021 TCTAGCAGCAGCTCCTCCAGCTCCAGCTCCAGCTCTGACTCATCTAGTTCCAGCTCTAGC

341 S S S S S S S S S S S S D S S S S S S S

1081 TCTAGCAGCAGCAGCTCCTCCTCCTCCTCTTCCAGCTCAAGCAGCAGCTCCGAAGAAGAC

361 S S S S S S S S S S S S S S S S S E E D

1141 CAATACTACAGAAGACACAACAACAACAACCGCAACAACAACAACAACAACAACAACAAC

381 Q Y Y R R H N N N N R N N N N N N N N N

1201 AACAACCGCAACAACAACCACAACAACAACAATAACGCCAACGACAACAACAATTCCGCC

401 N N R N N N H N N N N N A N D N N N S A

1261 AACAACAGCAACAACAACAACAGCAACAATTCCGCCAACAACAACAACAACAACAACAAG

421 N N S N N N N S N N S A N N N N N N N K

1321 AACAACAACCGCAACAATGACGAGAACGTCTCTCGCAGTCGCTCCCGCAGAGATATCTCC

441 N N N R N N D E N V S R S R S R R D I S

1381 CAATACAAATACAACAGTTTTGAGAACAACAACAACAACGACAACGAAGACGAAAAGAAT

461 Q Y K Y N S F E N N N N N D N E D E K N

1441 AACAGTGGCCGCAACGGACACAACGGACACAACGGAAACAACGGACACAACGGAAACAAC

481 N S G R N G H N G H N G N N G H N G N N

1501 GGACACAACGGACACAATGGACACAACGGAAACAATGGACAAAACAACAGCAATGGCAGC

501 G H N G H N G H N G N N G Q N N S N G S

1561 TCAAGCAGCAGCTCCAGCTCTAGCAGCTCTAGCTCTGGCTCCAGCAGCTCTGAAGAAAAC

521 S S S S S S S S S S S S G S S S S E E N

1621 AACTCCCGTTACAACAACGGTAAATTCGCCAGCTTTGCCCGCCACAACGGATCTGGATCC

541 N S R Y N N G K F A S F A R H N G S G S

1681 TCCTCCTCCAGCAGCTCCCCCGACTCTTCTGACTCCTCCAGCTCCTCCAGCTCATCCAGT

561 S S S S S S P D S S D S S S S S S S S S

1741 TCCTCCAGCTCCTCTAGCTCCTCCAGCTCTTCCAGCTCCTCATCCAGCTCTGAAGACAAC

581 S S S S S S S S S S S S S S S S S E D N

1801 AGCTCCTTTGGATCTTCCGTCTCCAGCAGCAGCGAAGAGGACTATGAACCACGTCCAAGC

601 S S F G S S V S S S S E E D Y E P R P S

1861 ACGTACAAGGCTCCTCAAACTCCTTTCTTCCCCTACTTCATCGGAAACTACGGTAACAGT

621 T Y K A P Q T P F F P Y F I G N Y G N S

1921 ATCCAATCCGCTAAACAAGTTAACGGAGTTGCTCTTGCCCGTAAGCTCGCCCAAGAAATT

641 I Q S A K Q V N G V A L A R K L A Q E I

1981 GCTGAGGAATTGAACGACCCACGTCAAATTACTCAAAAAAGCACTTTGGCTAAATTCAAC

661 A E E L N D P R Q I T Q K S T L A K F N

2041 ATGTTGGTTGAGGAACTCAGAACCCTGGACGCGAAACAAATGGAACAAGCTTCCCAAGAG

681 M L V E E L R T L D A K Q M E Q A S Q E

2101 CTTCATTTCAACTCCGCCCAGGCCTCCAGCCACAGCCGTCAAGATGCTCTGAAATCTCTC

701 L H F N S A Q A S S H S R Q D A L K S L

2161 GCCTGGAAATCCTTCTGTGATGCCTTAGTTGAAGCCGGTACCGGACCTGCCTTTTTGCAA

721 A W K S F C D A L V E A G T G P A F L Q

2221 ATCCAAAAGATCATTGAACACCAACAAGTCTCCGACGCCGAAGCCGCTCGCATGATCAGC

741 I Q K I I E H Q Q V S D A E A A R M I S

2281 CGTCTTCCAGTCACCGCTCGTTTCCCAGACAAGGAATACATGAACTCTTTCTTCAACTTT

761 R L P V T A R F P D K E Y M N S F F N F

2341 GTCAGATCCAACAATGTTCAACACCAGAACCAACTCAACGAAACTGCTCTCCTTGCTTTC

781 V R S N N V Q H Q N Q L N E T A L L A F

2401 GCTGAGCTTTGCCGTAAAGCCGATGTCAACGCCAGAAACGCCCACAACTACTACCCTGTC

801 A E L C R K A D V N A R N A H N Y Y P V

2461 CACGTTTATGGACGTGTCCTCCCAGAGCATGCCAAGGCTGTTGCTCACCAATACCTTCCC

821 H V Y G R V L P E H A K A V A H Q Y L P

2521 TACTATGAACAAAACCTCAAGAGAGCCGTTGCCAACGGTGACAGCCGCAAGATCCAAGCT

841 Y Y E Q N L K R A V A N G D S R K I Q A

2581 TACATCCGTGCCATTGGAAACTTTGCTCACCCCAAGATCCTCGAAGTTTTCGAGCCCTAC

861 Y I R A I G N F A H P K I L E V F E P Y

2641 CTTGAAGGAAAGGTCCCAATCTCCAACTTCCAACGCACTGTCATGGTCCTCTCCCTTAAC

881 L E G K V P I S N F Q R T V M V L S L N

2701 GAACTTGCACGCGTCTACCCCAACCTTGCCCGCAATGTCCTCTTCAAGATCTACCAAAAC

901 E L A R V Y P N L A R N V L F K I Y Q N

2761 ACCCAAGAAAACCAAGGAGTCCGTGTTGCCGCTGTCTTCTTAATCTTCGGAACCAACCCA

921 T Q E N Q G V R V A A V F L I F G T N P

2821 TCTGCCCAGACCCTCCAACGTATGGCTCAATTCACCAATGAAGACCAAGACCAGCAAGTC

941 S A Q T L Q R M A Q F T N E D Q D Q Q V

2881 AACGCTGCCGTCAAATCCGCCCTTGAAAACGCTGCCAAGGCCCACTCCGAGTCTCGTCAA

961 N A A V K S A L E N A A K A H S E S R Q

2941 GAACTCGCCCAAGCTGCTCAATCCGCTATTGCTCTCCTCAGCCCTAAGACTTACGGTCTT

981 E L A Q A A Q S A I A L L S P K T Y G L

3001 CAATACTCCAAGAAATGGCTCCGCGACTACATCGTCAAGGAAGAAAACCTTGCCTACAGA

1001 Q Y S K K W L R D Y I V K E E N L A Y R

3061 GTGTCTGCCGATATGATCCAGAGTGAGGACTCCTTAATCCCCAACCAAGTTTACGTTGCC

1021 V S A D M I Q S E D S L I P N Q V Y V A

3121 CTTCACCGTTACCTCGGTGGATTCGCTCAACGTGTTGCCAGCTTCAGAGCCATGACCTCC

1041 L H R Y L G G F A Q R V A S F R A M T S

3181 AGTGCCAGTGACCTCGTTGAAAAGATCCAAGAACAGTTCACCAATGGAGAAGAATACCAA

1061 S A S D L V E K I Q E Q F T N G E E Y Q

3241 CAACAATCCGAAATGAACCAGCAATTCTCTGCCGAACAAATCTTCAGACAGTTCAACATT

1081 Q Q S E M N Q Q F S A E Q I F R Q F N I

3301 AAGCCTGACTACCCACAAGAAGTTGAAGCCCTCCTCCAATACACCGTTTTCGGAGCCAAG

1101 K P D Y P Q E V E A L L Q Y T V F G A K

3361 CGCTGGGCCTTCTTCGACGAAGAATTCTTCAACCAAATCCCAAGACGTCTGAACGATGCT

1121 R W A F F D E E F F N Q I P R R L N D A

3421 CCCTCTAAGGTTCAAAATGGACAATCTTTCAACTCCACCAAATTCTACAACGACATCTCT

1141 P S K V Q N G Q S F N S T K F Y N D I S

3481 CTCTCCCTTGCTTTCCCCACTGCCACTGGTCTGCCATTCTCTTACACTCTCAAGGTCCCC

1161 L S L A F P T A T G L P F S Y T L K V P

3541 ACTCTCGTCCAAGCCGGTGGAGAAGTTCAAGCCAGAGTTCAAGGTCACAACTCCAACAAC

1181 T L V Q A G G E V Q A R V Q G H N S N N

3601 AACAACAACAACAACAACCTCTTCCGTATCCCTGAAGCTGTTAACGTAACCGCCGAAATT

1201 N N N N N N L F R I P E A V N V T A E I

3661 GAAATTGTTTACGCCACTGAATTGAAATCTGAACTTGGATTCGTCACTCCATTCAACCAC

1221 E I V Y A T E L K S E L G F V T P F N H

3721 GAACGTTACGTTGCTGGTCTTGCCAAGAACATCTTTGTTAACATCCCAGTTAAGGTCGCC

1241 E R Y V A G L A K N I F V N I P V K V A

3781 GCCAATGTGGACATCGCCAACACCAAGGTCGAATTCTACATGAAGCCTATGAACAACCAG

1261 A N V D I A N T K V E F Y M K P M N N Q

3841 AACGAACAAAAAATCTTCCACTACGGATCTTACCCTTACACCGCAATCCAAAACATCTTC

1281 N E Q K I F H Y G S Y P Y T A I Q N I F

3901 GACTTCCGCCCCCTCCAAGAAAACGAAAACACTAAGTACATCTTCGCCAACGAGAACAAG

1301 D F R P L Q E N E N T K Y I F A N E N K

3961 AACAAGTTCGAGAAAGTCTACGGTGAAGAAAAGACCGGTTTTGCCTTCCGTTGCCAATAC

1321 N K F E K V Y G E E K T G F A F R C Q Y

4021 AAGGGTGACCAACAAAGCTTCCAATTCGCTGACTTCTACAACTTCGCTAAGCGTAACGAT

1341 K G D Q Q S F Q F A D F Y N F A K R N D

4081 TTCTTCTCCGCTGCTTTCTTCCCATGGGCTGAAAAGACCATCCAATACAACAACTTCGAT

1361 F F S A A F F P W A E K T I Q Y N N F D

4141 GTCTACTATGACCCAGCTAGATCTGCCGCCAAATCCGCCAAGTTCGCCTTGAACTATGCC

1381 V Y Y D P A R S A A K S A K F A L N Y A

4201 AACAAATACGCCAACAAGGAAAACAACAACGAAGGTAACTCTAACAACCACAACAACAAC

1401 N K Y A N K E N N N E G N S N N H N N N

4261 CAAAACGATGCTGTTCCATCTTCCTACCAACCTGACAACGAACAGAGAATGAACCAATTC

1421 Q N D A V P S S Y Q P D N E Q R M N Q F

4321 GCCAGCCGTGCTCAATCTGGAGTTCAAAGTGCCAACATCGATGTTATCGGCATCTCTGCT

1441 A S R A Q S G V Q S A N I D V I G I S A

4381 CAATTCTTCGGACAAAAGAACGCTGATTACGTCGCCACTCTTGCTTACGCTCGTAGCCCA

1461 Q F F G Q K N A D Y V A T L A Y A R S P

4441 GTCGCTGAAAAGGCTCGTTTCCTCTTCTACGCCGGTGCTAACAATGCCAACAACAACAAA

1481 V A E K A R F L F Y A G A N N A N N N K

4501 AACAAGGTTGCTGTTGACGCCACCGCTTACATGCCCAATGTTCCTCTCGTCAACGCCGCT

1501 N K V A V D A T A Y M P N V P L V N A A

4561 CAAGCTTTCAACGCCGACGCCAACTCCCGCTTCTACGCCAACGTTAAATCTGGAGAAAAC

1521 Q A F N A D A N S R F Y A N V K S G E N

4621 CTCGACAATGGTGCTCAATTCCAATTCCAAGCTAATATGAAGCAAAGCCAAGAGTTCCGT

1541 L D N G A Q F Q F Q A N M K Q S Q E F R

4681 GACTACTTCCGCCAAAGCCAAATGTACAAACAGTGCTCTCAACAAATGGAACAAGGAGAA

1561 D Y F R Q S Q M Y K Q C S Q Q M E Q G E

4741 TACATGATGCCTGCTTGCCGTAACGCTACCGTTGTCGCTAACAGATTAAACGAAGCCCAC

1581 Y M M P A C R N A T V V A N R L N E A H

4801 TTCTCCATCAACTTTGACAAGGTTTCTGATGCCGTCAAGAACTACACTTACCAAGCTTTC

1601 F S I N F D K V S D A V K N Y T Y Q A F

4861 GCCTACGCCCGTCATTTAGGATACCAATACCAGTCTGAAAACTTCGGAAACCCCAATGGC

1621 A Y A R H L G Y Q Y Q S E N F G N P N G

4921 CAACACAACAAGATTGATGGTTACTTCAAGTTCTCCCCTAAGTTCGACTTCGCCCAGTTC

1641 Q H N K I D G Y F K F S P K F D F A Q F

4981 TACTTCAACGCTCCTTCCGTAGCCGCTTCCTTCAAGAACGTTCCAGTTCACCAATACGTC

1661 Y F N A P S V A A S F K N V P V H Q Y V

5041 GCTGACTTTTTCGCCCCCCACCCCGTCTACTCTGGCTTCGACCGTCTCATGCAAGACACT

1681 A D F F A P H P V Y S G F D R L M Q D T

5101 TTCCAAGCCAAATACCAAGCTGCTTGCGTTGCTGACAAGATGCACGCTACCACTTTTGAC

1701 F Q A K Y Q A A C V A D K M H A T T F D

5161 AACAAAACCTACCCCCTTCACATGCAACAAAACAACTGGTACGTCCTCATGACCTACGTC

1721 N K T Y P L H M Q Q N N W Y V L M T Y V

5221 AACAGGAACAACTACTACAACAACCAATACAACAGCTACTTTCAAGGAAACAATAAGAAC

1741 N R N N Y Y N N Q Y N S Y F Q G N N K N

5281 CAATACAGCTACAGAGACTACAACCAAAAGAGATTCTACACTACCGTCTATGCTAGACAA

1761 Q Y S Y R D Y N Q K R F Y T T V Y A R Q

5341 AATAGCAACGGCCAAAAGGAATTGAAAATTGTCCTGAACAACGGAGAATACGAAATCTTC

1781 N S N G Q K E L K I V L N N G E Y E I F

5401 ATGCAACCAGCCTCCTCTCGAGCCGGTCTCCACAGCTCTAACTCCGGAAAGAACAACGCT

*

*

*

*

*

*

*

*

*

1801 M Q P A S S R A G L H S S N S G K N N A

5461 GCCATCAAGGTCTTCATCAACAAACAAGAACAACAATTCAACGACAAGCAATTCACTGAC

1821 A I K V F I N K Q E Q Q F N D K Q F T D

5521 TTCCACGGACACAATGGAAAGATCTACGCTCAATTCTACGCTCTTCCTGATGGTGCTATC

1841 F H G H N G K I Y A Q F Y A L P D G A I

5581 CGCTTCTTCGCTCCCCAATCTGGACTTCAAGCCATCTATGATGGAGCTCGCATCAAGATT

1861 R F F A P Q S G L Q A I Y D G A R I K I

5641 CAAGCTGCCAACCAATACAGAGGTGCTGTCCGTGGTATGTGCGGAACCTACTCCAACCAA

1881 Q A A N Q Y R G A V R G M C G T Y S N Q

5701 TACGCTGACGACTTCACCGCCCCTCAAAACTGTGTCTACAAGAACCCAGAAGACTTCGCT

1901 Y A D D F T A P Q N C V Y K N P E D F A

5761 GCCGTCTACGCTGTCATTGACTCTTCTTCCCCCTCTCAAGTGAAATCCCAGAAGGAACGC

1921 A V Y A V I D S S S P S Q V K S Q K E R

5821 GCTCAGCAAAACTTCTGTGCCCACAAGAACAACCAATTCGGAAACTACGTTAGCCGCAGC

1941 A Q Q N F C A H K N N Q F G N Y V S R S

5881 GACGCCGGATACGGATACAAATACAACAACAACGACAAATACTACGAATCCGCCTACAAG

*

1961 D A G Y G Y K Y N N N D K Y Y E S A Y K

5941 AACACCAAATACTACGACCCCTCCAACTCCCAGTACAACAAGTACTACAAGAACAACAAG

1981 N T K Y Y D P S N S Q Y N K Y Y K N N K

6001 TACGCCAAGGACCAGGACTCCTCCTACTCCAGCAGCAGCTCTTCATCTAGCGACTCTAGC

2001 Y A K D Q D S S Y S S S S S S S S D S S

6061 AGCAGCTCATCCAGTGACTCCAGCAGCAGCTCCTCTTCTTCCTCCTCCAGCTCATCCAGC

2021 S S S S S D S S S S S S S S S S S S S S

6121 TCTAGCAGCGACTCCAGCTCTTCCCAGTCCAACGACAACAACCGCAACAACAACAACCGC

2041 S S S D S S S S Q S N D N N R N N N N R

6181 AACAACAACAACCGACAACAACAACAACAACAACCGCAACAACAACAACAACCGCAACAA

2061 N N N N R Q Q Q Q Q Q P Q Q Q Q Q P Q Q

6241 CAGCAACAACAACAACAACCGCAACAACAACCGCAACAACAACCGCAACAACAGCAACAA

2081 Q Q Q Q Q Q P Q Q Q P Q Q Q P Q Q Q Q Q

6301 CAACAACAGCAGCAGCCAAGAAGCCAGTTACGAACAGAGAAACCAAAACGGACCCTCTAT

2101 Q Q Q Q Q P R S Q L R T E K P K R T L Y

6361 CCGCAAACTGTACCGTGCCATCAACCAAGGAGACGACATGTGCTTCACCATCAACGCTAT

2121 P Q T V P C H Q P R R R H V L H H Q R Y

6421 CCCAACTTGCAGATACCCAGCCAAGCCCGTTGGAAGCGCCAAGAAAATGGTTGA

2141 P N L Q I P S Q A R W K R Q E N G *
