# Supplementary material for: Enhanced Vitellogenesis in a Whitefly via Feeding on a Begomovirus-Infected Plant
Source: PLoS One. 2012 Aug 24;7(8):e43567. doi: 10.1371/journal.pone.0043567 (PMC3427354; doi:10.1371/journal.pone.0043567)
Supplement: Information S1 — Details of methods, sequences of vitellogenin cDNA. (DOC) [file pone.0043567.s007.doc]

**Enhanced vitellogenesis in a whitefly via feeding on a begomovirus-infected plant**

**Jian-Yang Guo^1#^, Sheng-Zhang Dong^2#^, Xiu-ling Yang^3^, Lu Cheng^1^, Fang-Hao Wan^4^, Shu-Sheng Liu^1^, Xue-ping Zhou^3^, Gong-Yin Ye^1^**^*^

1 Ministry of Agriculture Key Laboratory of Agricultural Entomology, Institute of Insect Sciences, Zhejiang University, Hangzhou, China

2 College of Life Sciences, China Jiliang University, Hangzhou, China

3 State Key Laboratory of Rice Biology, Institute of Biotechnology, Zhejiang University, Hangzhou, China

4 State Key Laboratory for Biology of Plant Diseases and Insect Pests, Institute of Plant Protection, Chinese Academy of Agricultural Sciences, Beijing, China

* Corresponding author address: Institute of Insect Sciences, Zhejiang University, 866 Yuhangtang Road, Hangzhou 310058, China. Phone: (+86) 571-88982696. Fax: (+86) 571-8795124. E-mail: [chu@zju.edu.cn](mailto:chu@zju.edu.cn)

# These authors contributed equally to this work.

**This file includes:**

Materials and Methods

Supplementary References

Supplementary Figures S1-S6

**MATERIALS AND METHODS**

**Whitefly**

The MEAM1 (mtCO1 GenBank accession no: GQ332577), MED (mtCO1 GenBank accession no: DQ473394) and Asia II3 (mtCO1 GenBank accession no: DQ309077) cryptic species of the whitefly species complex *Bemisia* *tabaci* (Gennadius) were used [1-3]. Stock whitefly cultures were maintained on cotton (*Gossypium* *hirsutum* L.) cv. Zhemian 1973 in separate climate chambers at 26ºC (± 1 ºC), 40–60 % RH and L: D 14 h: 10 h light regime. The purity of the cultures was checked every 3-5 generations using a random amplified polymorphic DNA-polymerase chain reaction (RAPD-PCR) [1, 4]. Measures were taken to use only pure sub-cultures of the MEAM1, MED and ASIA II3 for experiments. Non-viruliferous whitefly colonies were reared separately under the same conditions as described above.

**Virus inocula**

Infectious clones of TYLCCNV and their satellite DNA molecules (named TYLCCNB) constructed previously [5, 6] were used as inocula, and the viruses were maintained on plants of tobacco (*Nicotiana tabacum* L.) cv. NC89 at 26ºC (± 1 ºC), 40–60 % RH and L: D 14 h: 10 h light regime.

**Plants**

Tobacco cv. NC89, a host plant of TYLCCNV, and a non-host plant, cotton (cv. Zhemian 1973), were used. Uninfected tobacco and cotton plants were grown under natural light and controlled temperature in insect-proof greenhouses [3]. The virus-infected tobacco plants were obtained by inoculating with TYLCCNV and TYLCCNB at the 4-5 true-leaf stage as previously described [5-7]. Both uninfected and TYLCCNV-infected tobacco plants were grown to the 6-7 true-leaf stage for experiments. The virus infection status of the test plants was judged by the characteristic symptoms, further confirmed by molecular markers as previously described [3]. All plants were watered every 3-4 days as necessary and fertilized once a week. All experiments were conducted at 26ºC (± 1 ºC), 40–60 % RH and L: D 14 h: 10 h light regime.

**Virus purification**

*Nicotiana benthamiana* plants at 30 days post inoculation with TYLCCNV and TYLCCNB were used as the source for virus purification. The process of virus purification was modified from one of our previous studies [8]. Leaf and stem tissues were frozen in liquid nitrogen and homogenized using ice-cold buffer containing 500 mM sodium phosphate, pH7.5, 1 mM EDTA, and 0.1% (v/v) 2-mercaptoethanol. The extract was squeezed through four layers of muslin, followed by a 20-min centrifugation at 6,000 g. The supernatant was clarified by the addition of 2.5% Triton X-100, 0.1 M sodium sulfite, and 10% (v/v) cold chloroform followed by a 10-min centrifugation at 8,000g. The supernatant was stirred overnight with 7% PEG6000, and centrifuged at 11,000 g for 15 min. The pellets were resuspended thoroughly in 0.5 M sodium phosphate containing 0.01 M magnesium chloride and 0.5 M urea. Virus suspension was layered on top of 20-30% sucrose cushion and centrifuged at 3,3000 g for 2 h. Pellets were suspended in 0.01 M phosphate buffer and used for further analysis. The virus was further confirmed by PCR using the method previously published [6]. Inactivated viron was acquired by sterilizing at 121 ºC for 20min and stored at 4 ºC before being used.

**Egg samples**

Newly laid eggs (2-3 h old) of MEAM1 were brushed from the surface of cotton leaves and collected into a sterilized Eppendorf microcentrifuge tube in an ice bath. To extract vitellin, the eggs were homogenized with a glass rod in 100 μl phosphate buffered saline (PBS, pH 7.4) with 2 μl of a protease cocktail containing 0.1 M phenylmethyl sulfonyl fluoride (PMSF) (AMRESCO). Subsequently, the mixture was centrifuged at 12,000 g for 30 min at 4 ºC; the supernatants were collected and the protein concentration was determined with the Bio-Rad protein assay kit (Bio-Rad, USA) using bovine serum albumin (BSA) as a standard. And then, the supernatants were stored at -70 ºC before being used.

**Gel electrophoresis**

**Characterization of vitellin**

Native-polyacrylamide gel electrophoresis (PAGE) was carried out on a 4-20 % gradient gel with a 4 % spacer gel (Bio-Rad). After electrophoresis, proteins were stained with Coomassie brilliant blue R-250. To test the presence of carbohydrate, lipid and phosphorus components, the gels were stained using GelCode® glycoprotein (Pierce, USA), Sudan black B and GelCode® phosphoprotein (Pierce, USA) respectively, following the methods described in the kit protocol.

**Characterization of vitellogenin**

SDS-PAGE gel electrophoresis was preformed with 8 % separation gel and 4% spacer gel. The gels were run at 100 V for 2 h at 4 ºC with a Mini-PROTEAN® 3 Electrophoresis cell (Bio-Rad, USA), and visualized with Coomassie brilliant blue R-250. The gel was photographed and analyzed using Gel Dom^TM^ XR+ Imaging system (Bio-Rad, USA).

**Western blot**

After electrophoresis, proteins separated on native-PAGE or 8 % SDS–PAGE gels were blotted onto nitrocellulose membrane in buffer C (25 mM Tris, pH 8.3; 192 mM glycine; 10% methanol) at 16V for 20 min, running on a Trans-Blots SD semi-dry transfer cell (Bio-Rad). The membrane was subsequently incubated with the purified monoclonal antibodies against MEAM1 Vt at 1:5,000 dilution for 60 min at 37ºC, followed by incubation with horseradish peroxidase (HRP)-conjugated goat anti-mouse IgG (Sigma) at 1:10,000 dilution for 60 min at 37ºC. The color was developed using 3, 3, 5, 5-Tetramethylbenzidene (TMB) (Promega) [9].

**Preparation of antibodies**

**Polyclonal antibody**

Following the method of Dong et al. [9], rabbit serum was prepared against Vt. The serum was made female specific by absorbing whole male homogenate solution using the method of Wu and Ma [10] and was purified by a PROSEP-A spin columns (Millipore). The serum titer had an enzyme linked immunosorbent assay (ELISA) end point of 1:68,000 by indirect ELISA. The female specificity to Vg was verified using Western blotting analysis.

**Monoclonal antibody**

The purified Vt with a final concentration of 1 mg/ml were used as antigen for preparing monoclonal antibodies following the method described by Dong et al. [9]. The hybridomas were screened for production of Vt-specific antibody using indirect ELISA and Western blotting. All the cell lines were propagated for ascites production and liquid nitrogen vapor phase cryogenic storage.

**Purification of antibodies**

To obtain purified monoclonal antibody, 4 ml of ascites were mixed thoroughly with an equal volume of saturated ammonium sulfate solution overnight at 4 °C, centrifuged at 10,000 g for 10 min. The precipitate was dissolved in 2ml PBS and dialysed at 4 °C with the same buffer for 24 h. Following dialysis, insoluble material was removed by centrifuge at 10,000 g for 10 min. The supernatant was applied to UNOTM Q1 ion exchange column (Bio-Rad) which had been equilibrated in buffer B (10 mM Tris-HCl, pH 8.2). The column was eluted with a linear NaCl (0.1-1 M) in buffer B (pH 8.2). The purity of the antibody was identified by SDS–PAGE. In addition, the immunoglobulin subclass of the antibody was determined using a goat anti-mouse IgG (H+L chain specific) kit (Southern Biotech, USA) by indirect ELSA.

**Sequencing of Vitellogenin cDNA**

**RNA isolation and synthesis of the first stand cDNA**

Total RNA was extracted using Trizol Reagent and further purified with the RNeasy-Mini Kit (Qiagen). Briefly, two hundreds of ovaries of adult females were dissected individually by tearing the epidermis in a drop of cold DEPC buffer. They were then collected into RNAse free glass tube for isolating total RNA. The RNA samples were quantitated using a Nanodrop spectrophotometer (Nanodrop Technologies, USA). The first strand cDNA were synthesized reverse transcribed in 20 μl reaction mixtures containing reaction buffer, oligo (dT)_18_ (10 mM), dNTP mixture (10 mM) and reversetranscriptase from avian myeloblastosis virus (AMV, 20 units) (Takara, Japan). The cDNA were then used as a template to generate target gene from an in vitro PCR.

**Primers and contigs**

Based on the published partial amino-acid sequences of *B*. *tabaci* Vg [11] and conserved sequence of other Hemiptera insects, two pairs of degenerate primers including Vg-sense primer1 (5’-ACN GGY GAY TGY GAR AC-3’), Vg- sense primer 2 (5’-ACA RAA AGC YGA RGT HYA CAG-3’), Vg-Reverse primer 1 (5’-GTR DGT CAT TTC AGC CAT-3’) and Vg- Reverse primer 2 (5’-GGC ATR TTR TTR GGR TTY TG-3’) were designed. Vg contigs were acquired after the PCR and assembled with an overlap of at least 200 base pairs.

**Rapid amplification of cDNA ends**

Based on cDNA sequence data obtained from the PCR product, the gene specific primers VgR1 (5’-TAG CCA TTT GTT TAG TCG TT-3’) and VgR2 (5’-CTG GGT TTC AGC ATT ATT CAG GTG TTC-3’) were synthesized for 5’-RACE; VgS1 (5’-TAC TCG GTA ACG ACT AAA CAA A-3’) and VgS2 (5’-CTT CAG GAT ATG GCT CAA CA-3’) were synthesized for 3’-RACE. The amplification conditions used were 5 min at 94°C, followed by 33 cycles of 30 s at 94°C, 30 s at 55°C, and 2 min at 72 °C, then 10 min at 72 °C for 5’-RACE; 5 min at 94°C, followed by 33 cycles of 30 s at 94°C, 30 s at 58°C, and 5 min at 72°C, then 10 min at 72°C for 3’-RACE. All of the above were followed instructions of the smart RACE Kit (Clontech, America). After PCR, products were separated by electrophoresis, DNA bands corresponding to approximately 1300 bp (for 5’-RACE) and 4500 bp (for 3’-RACE) were excised from the agarose gel and purified using a DNA gel extraction kit (Omega, USA). These PCR products were cloned into the pGEM-T-easy vector (Promega, USA) and sequenced by the dideoxynucleotide method. To confirm the validity of the sequence data obtained, each fragment was sequenced at least three times. The overlapping sequences from PCR were assembled to obtain a full-length cDNA sequence of one subunit of Vg in MEAM1.

**Deduced amino acid sequence and phylogenetic analysis of Vg genes.**

The sequence of MEAM1 Vg cDNA was compared with those of other Vg sequences deposited in GenBank using the ‘‘BLAST-N’’ or ‘‘BLAST-X’’ tools available on the National Center for Biotechnology Information (NCBI) website. The amino-acid sequence of MEAM1 Vg was deduced from the corresponding cDNA sequence using the translation tool at the ExPASy Proteomics website (http:// www.expasy.org/tools/dna.html). Other protein sequence analysis used in this study, including molecular weight (MW), isoelectric point (PI) and multiple sequence alignments of deduced amino-acid sequence, was performed using MEGA 4.0 software [12]. The position of the signal peptide in the Vg was predicted using the Signal IP computer program on the website: http://www.cbs.dtu.dk/services/SignalP.

**Supplementary References**

1. Xu J, De Barro PJ, Liu SS (2010). Reproductive incompatibility among genetic groups of *Bemisia tabaci* supports the proposition that the whitefly is a cryptic species complex. Bull Entomol Res 100: 359-366.
2. Liu J, Zhao H, Jiang K, Zhou XP, Liu SS (2009). Differential indirect effects of two plant viruses on an invasive and an indigenous whitefly vector: implications for competitive displacement. Annals Appl Biol 155: 439-448.
3. Jiu M, Zhou XP, Tong L, Xu J, Yang X, et al. (2007). Vector-virus mutualism accelerates population increase of an invasive whitefly. PLoS One 2: e182.
4. Zang LS, Liu SS, Liu YQ, Chen WQ (2005). A comparative study on the morphological and biological characteristics of the B biotype and a non-B biotype (China-ZHJ-1) of *Bemisia tabaci* (Homoptera: Aleyrodidae) from Zhejiang, China. Acta Entomol Sin 48: 742-748.
5. Li ZH, Xie Y, Zhou XP (2005). Tobacco curly shoot virus DNAβ is not necessary for infection but intensifies symptoms in a host-dependent manner. Phytopathol 95: 902-908.
6. Cui XF, Tao XR, Xie Y, Fauquet CM, Zhou XP (2004). A DNAβ associated with tomato yellow leaf curl China virus is required for symptom induction. J Virol 78: 13966-13974.
7. Zhou XP, Xie Y, Tao XR, Zhang ZK, Li ZH (2003). Characterization of DNA beta associated with begomoviruses in China and evidence for co-evolution with their cognate viral DNA-A. J Gen Virol 84: 237-247.
8. Zhou XP, Chen JS, Li DB, Li WM (1994) A method of purification of potyviruses with high yield. Chinese Microbiol 21: 184–186.
9. Dong SZ, Ye GY, Zhu JY, Chen ZX, Hu C, et al. (2007). Vitellin of *Pteromalus puparum* (Hymenoptera: Pteromalidae), a pupal endoparasitoid of *Pieris rapae* (Lepidoptera: Pieridae): Biochemical characterization, temporal patterns of production and degradation. J Insect Physiol 53: 468-477.
10. Wu SJ, Ma M (1986). Hybridoma antibodies as specific probes to *Drosophila melanogaster* yolk polypeptides. Insect Biochem 16: 789-795.
11. Leshkowitz D, Gazit S, Reuveni E, Ghanim M, Czosnek H, et al. (2006). Whitefly (*Bemisia tabaci*) genome project: analysis of sequenced clones from egg, instar, and adult (viruliferous and non-viruliferous) cDNA libraries. BMC Genom 7: 79.
12. Tamura K, Dudley J, Nei M, Kumar S (2007). MEGA4: molecular evolutionary genetics analysis (MEGA) software version 4.0. Mol Biol Evol 24:1596-1599.

**Supplementary figure legends**

**Figure S1 Characterization of MEAM1 whitefly Vt.**

Native-PAGE (linear gradient consisting of 4–25% polyacrylamide) analysis with Coomassie brilliant blue staining (left) and the characterization of yolk soluble protein (right), Vt bands were visualized by staining with Coomassie brilliant blue (C), Sudan Black B (L), Periodic acid-Schiff’s reagent (P) and Methyl Green Solution (G). The soluble proteins sampled from eggs (E), ovaries (O), female (F) and male (M) adults 6 d after eclosion. PM: high molecular weight standards (Amersham). Arrows indicate the bands of Vg or Vt.

**Figure S2 Purification of monoclonal antibody against MEAM1 whitefly Vt.**

SDS–PAGE of the purified monoclonal antibody IgG against *B*. *tabaci* Vt with Coomassie brilliant blue staining. PM: Molecular weight standards; A: purified IgG; B: crude ascites fluid; H and L: IgG heavy and light chain.

**Figure S3 Distribution of Vg/Vt in different tissues of MEAM1 whitefly.**

SDS–PAGE analysis with Coomassie brilliant blue staining (left) and corresponding Western blotting analysis (right) with the monoclonal antibody against *B*. *tabaci* Vt for soluble proteins sampled from different tissues of the female and male. PM: prestained molecular mass markers (Bio-Rad); E: egg extract; H and O: female hemolymph and ovaries 6 d after eclosion; F and M: soluble protein of female and male adults 6 d after eclosion; Arrow indicates subunits of Vg or Vt.

**Figure S4 Immune reaction of Vt antibody with yolk protein of MED and ASIA II3 whiteflies.**

SDS–PAGE analysis with Coomassie brilliant blue staining (left) and corresponding Western blotting analysis (right) with the monoclonal antibody against *B*. *tabaci* Vt for soluble proteins sampled from MEAM1, MED and ASIA II3 whiteflies. PM: prestained molecular mass markers (Bio-Rad); BF, QF and ZF: soluble protein of MEAM1, MED and ASIA II3 female adults 6 d after eclosion. BM, QM and ZM: soluble protein of MEAM1, MED and ASIA II3 male adults 6 d after eclosion; Arrow indicates subunits of Vg or Vt.

**Figure S5 Nucleotide and deduced amino acid sequence of the vitellogenin cDNA of MEAM1 whitefly, *Bemisia tabaci*.** “ ” signal peptide, “ ”functional motif, “ ”polyserines, “ ”unknown motif

**Figure S6 Phylogenetic tree of Vg in MEAM1 *Bemisia tabaci* and other insects of their predicted amino acid sequences using the neighbor-joining method.**
